# Estimating Effects of Radiation Frost on Wheat Using a Field-Based Frost Control Treatment to Stop Freezing Damage

**DOI:** 10.3390/genes13040578

**Published:** 2022-03-24

**Authors:** Brenton A. Leske, Thomas Ben Biddulph

**Affiliations:** 1 The Department of Primary Industries and Regional Development, 3 Baron Hay Court, South Perth, WA 6151, Australia; brenton.leske@dpird.wa.gov.au; 2 The School of Agriculture and Environment, The University of Western Australia, 35 Stirling Highway, Crawley, WA 6009, Australia

**Keywords:** *Triticum aestivum* L., frost, floret sterility, phenotyping, diesel heater, frost control

## Abstract

Crop phenotyping experiments have long struggled to have a reliable control treatment that excludes frost and associated freezing damage to plants. Previous attempts used a barrier, such as a removable shelter or cloth to exclude frost. However, these methods were labour intensive and varied in their effectiveness. An automated diesel heater was used to protect field plots of wheat (*Triticum aestivum* L.) from frost damage. In 2018 and 2019 there were 22 and 33 radiation frost events from July to October at the field site. The heater maintained canopy air temperature above freezing (>0 °C) for the duration of the frost (~6–8 h). Heated plots had 2–3 °C warmer minimum canopy air temperatures. Cold and chilling damage was still present in heated plots and represented 20–30% floret sterility; freezing damage in non-heated plots accounted for an additional 10–30% floret sterility. Grain mapping revealed: grain set in the apical spikelets is most affected by frost damage; proximal florets (G1 and G2) contribute the most to grain yield, but distal (G3 and G4) are important contributors to grain yield when sterility in proximal florets occurs. These results demonstrate that a plot heater is a useful tool to study frost-induced freezing damage in cereal crops, by way of preventing freezing damage in heated field plots for direct comparison to naturally frosted plots. This approach could be used to develop improved damage functions for crop simulation models through a dose and timing-response experiment for natural frost incidence on cereal crops in field plots.

## 1. Introduction

Sub-zero canopy air temperatures at the reproductive stage of cereal crops cause considerable damage to plants, reducing grain yields and profitability of farming in southern Australia and other frost-prone regions throughout the world [1,2,3,4]. Frost can occur in two different forms: advection frost and radiation frost. Advection frost results from the incursion of cold air masses [5,6]. Radiation frosts occur on cloudless, cold nights, where longwave radiation radiates towards the sky, resulting in a net cooling of the crop canopy as heat is lost to space [7,8]. Frosts in Australia were thought to be predominantly radiation frost; however, recent research has discovered there is an advective component to radiation frosts in Australia in which dry, cold polar air moves from Antarctica and descends on the southern regions of the grainbelt in Western Australia, South Australia (SA), Victoria (VIC) and southern New South Wales (NSW) [9]. Radiation frost after the advective cooling of the landscape is the predominant type of frost that occurs in late winter and early spring in WA [7,9], and is what will be referred to by the term “frost” from hereon.

Frost can cause damage to wheat at pre spike emergence, stem elongation and post spike emergence [1,10,11]. During frosts, there are three types of temperature-related damage to wheat plants: cold (<8 °C), low temperature/chilling (0–5 °C) and freezing (≤0 °C) damage [12,13,14,15]. Each of these types of temperature-related damage affects wheat plants in different ways. Cold and chilling temperatures adversely affect pollen viability in wheat, with the severity of damage increasing with decreasing temperatures [16]. The freezing of plant tissue causes dehydration and damage to cell membranes [7,17,18]. Recent research has shown that wheat plants in the field freeze from the ground up, with older and senesced leaves freezing first; individual tillers froze in a random order [19]. Further understanding of the freezing process in the field during frosts is needed to explain differences in cultivar performance under frost conditions at the reproductive stage [18,19,20,21]. Moderating the effect of frost in field experiments, to provide “frost-free” controls to compare against the frost-exposed plots, is critical to understanding the temperature-related effects of frost on wheat plants during and after frost events. 

Crop models such as the Agricultural Production Systems Simulator (APSIM) [22] have been used to identify the optimal anthesis periods for wheat across Australia, accounting for soil water, radiation, heat and frost risk [23]. APSIM has been used to determine the economic cost of frost damage to growers in different grain-growing regions of Australia and the economic benefit of breeding less susceptible to frost damage cultivars [24]. The lack of frost controls in field trials has meant there is a lack of data on the impact of frost on wheat plants at various development stages. This has meant frost damage functions in models like APSIM have been based on limited data and best bet guesses [25,26], rather than field data with controls for frost damage treatments. Frost damage data which include “frost-free” controls would be useful in developing improved frost damage models. Models with an improved frost damage function based on data can then be used to more accurately model future climate scenarios and determine the economic cost of frost damage in Australia [24,27,28,29,30] and to optimise agronomic management decisions [23,31]. 

Efforts have been made to develop “frost-free” controls by moderating or preventing the effect of frost on experimental plots of cereal plants [3,4,5]. These attempts used a cover placed over plants, which is labour intensive and requires active monitoring. Fredericks et al. [3] used a cover that was automated but stated it was not reliable. Moreover, a limitation of frost covers can be shading of plants if not removed shortly after dawn [4,32,33]. Stutsel et al. [34] presented an automated plot heater to reduce frost exposure post spike emergence in wheat plots. This previous research demonstrated the effectiveness of the heaters to raise plot canopy temperatures during cold nights and validated that the heater did not notably impact plant growth or grain yield in the absence of frost. However, due to only mild frosts occurring during this study in 2017 (floret sterility was ≤10% across the five time of sowing blocks used in the field experiment), the impact of higher amounts of frost damage (20–80%) on grain yield and yield components compared to the non-frosted control could not be assessed. Since there are no published results using this plot heater in the field during frost events that caused >10% floret sterility, further novel experiments were conducted to validate the plot heaters to provide “frost-free” control plots under natural frost conditions ≤ 0 °C in 2018 and 2019 at the post spike emergence stage in wheat. The term “frost(s)” is defined here as air temperature ≤0 °C at canopy height, which is approximately equivalent to 2 °C air temperature in a Stevenson screen 1200 mm above ground level (AGL) [29]. 

The aim of this study was to quantify frost damage in wheat by comparison of plots in ambient natural frost conditions in a frost-prone environment to those with heaters that prevent frost in selected plots. The effectiveness of the plot heaters was evaluated by measuring: canopy air temperatures at three different heights, with further observations made by handheld thermography; the sterility in the proximal florets of the spike; grain yield and its components were recorded. Data are presented from two field seasons (2018 and 2019) to test the repeatability and reliability of the plot heaters to maintain canopy air temperatures above 0 °C in heated plots when the ambient air temperature falls below 0 °C.

## 2. Materials and Methods

### 2.1. Experimental Design

Field experiments were conducted at the Department of Primary Industries and Regional Development research site at Dale, Western Australia, over the 2018 and 2019 growing seasons (32°12′24.48″ S, 116°45′31.32″ E). The average elevation of the trial site was 245 m above mean sea level. The site is historically frost-prone, located on a flat valley with a 0.66% slope from the southwest to the northeast side of the trial site [8]. Growing season rainfall (April to October) in 2018 and 2019 was 346 and 322 mm respectively. 

The cultivar Wyalkatchem was chosen for the heated treatment because it represented a more frost-damage-susceptible wheat cultivar compared to other cultivars grown across the wheat-growing areas of southern Australia [21,35] and is used as a parent in breeding crosses for various modern Australian wheat cultivars [36]. To expose plant material to natural frost, each year a trial with eight times of sowing (ToS) blocks were established based on a predicted equidistant thermal time of ~250 growing degree days from early April to early June. This was done so that wheat would be at anthesis from July to October; the frost window for the region is from August to September. Two ToS blocks out of the eight were selected each year to deploy the plot heaters into, with the crop selected being not further advanced than Z39 at the time of deployment [37]. Further details of the deployment of the plot heaters are described in Section 2.2 “Plot heaters”. The soil was a grey sandy loam (pH 5.4 CaCl_2_ 0–10 cm). To reduce the confounding effects of stubble in altering frost damage [38], stubble was burnt before sowing. One 20 mm application of irrigation was applied two weeks before sowing to ensure germination occurred. The field plots (1.7 × 5 m) of wheat (*cv.* Wyalkatchem) consisted of six rows, 250 mm apart, which were sown at 75 kg ha^−1^ to achieve a target plant density of 150 plants m^−2^. Each ToS block was fertilised at sowing with 12 kg N ha^−1^ as a compound fertiliser (Gusto Gold, Summit Fertilizers, Kwinana, WA, Australia) and 23 kg N ha^−1^ as urea. Two additional applications of liquid fertiliser at 19 kg N ha^−1^ were applied at the tillering and booting stage as urea ammonium nitrate. 

**Figure 1:**
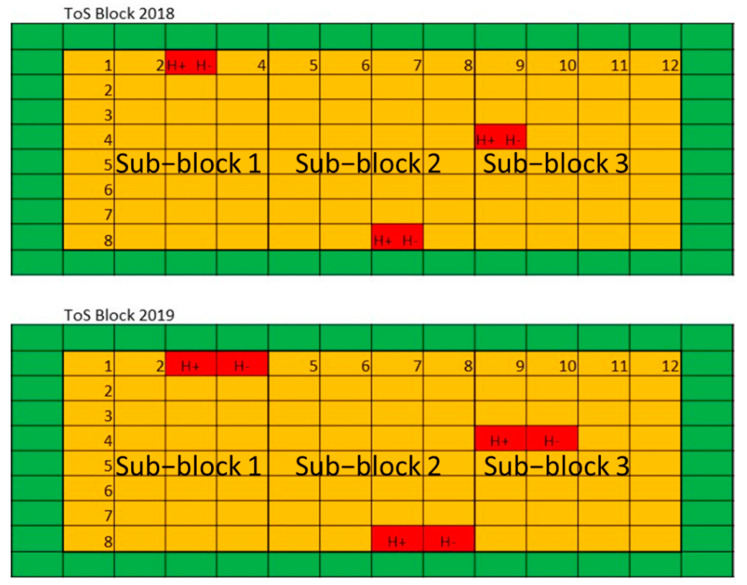
The field layout of the experiment displaying a model time of sowing (ToS) block from each year (2018 and 2019). Each ToS block contained three sub-blocks, with one plot replicate in each of these sub-blocks for all the 15 cultivars (two plots of each, including the heated and non-heated treatments for one cultivar, Wyalkatchem). Heated and non-heated treatments (red boxes) were located within each of the Wyalkatchem field plots within each sub-block. Buffer plots surrounded each ToS block (green boxes). Note that the location of heated and non-heated plots is not their actual location in the trials.

Individual plots were arranged in a randomised block design with three plot replicates (sub-blocks) per ToS block (main-block) (Figure 1). This design used the same approach as Cocks et al. [21]. ToS blocks were not randomised and as such were treated as separate environments in data analysis. Each ToS was analysed separately. Within the ToS block, heated and non-heated treatments were randomised amongst 15 other cultivars of wheat which were optimised using the DiGGer statistical software package [39]. The other cultivars were used for another experiment and will not be reported on in this paper but were included in the trial design to improve the randomisation and reduce spatial temperature variation across the ToS block [8,40]. These experiments used plot heaters as controls (Heater+) (i.e., plots in which frost was prevented) for the ambient frost treatment (Heater−). The plot heaters were initially developed and described in Stutsel et al. [34] (named prototype three). 

### 2.2. Plot Heaters

**Figure 2:**
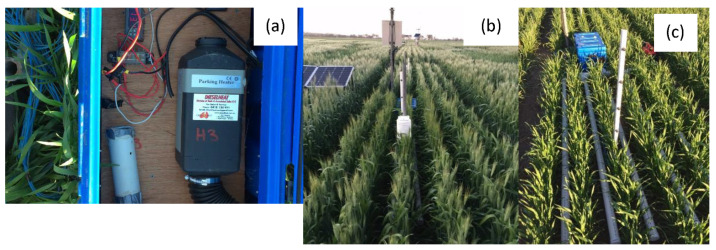
A 2 kW diesel plot heater (Belief, Diesel Heat, TAS Australia) housed in a waterproof case (**a**); the weather station (dataTaker DT50-S3, Lontek, NSW, Glenbrook, Australia) which recorded humidity (Vaisala Humitter 110 with a Humicap sensor, Lontek, NSW, Glenbrook, Australia) and canopy air temperature (T-type, Temperature Controls Pty. Ltd., Sydney, NSW, Australia) in non-heated and heated plots and activated the heater (**b**); the unshielded thermocouples which measured canopy air temperature and the heated plot area of wheat (*cv*. Wyalkatchem) (**c**).

Our goal was to test the repeatability and reliability of the plot heaters across different seasons to maintain canopy air temperatures above 0 °C in heated plots when the ambient air temperature falls below 0 °C. Briefly, a 12 V 2 kW diesel space heater (Belief 2 kW diesel space heater, Diesel Heat, Cygnet, TAS, Australia) was installed in a waterproof case (Pelican™ Products Pty Ltd., Erina, NSW, Australia) and hot air produced by the heater was distributed through a 1 m (L) × 1.2 m (W) PVC manifold (Figure 2a,c). The manifold was engineered to lay flat on the ground between the crop rows on five inter-rows and release hot air in a vertical direction via holes on the skyward facing surface of the pipes. The skyward facing surface of the pipes was 50 mm from the soil surface and the downwards facing surface of the manifold pipe was in direct contact with the soil surface. The manifold would heat the soil surface as well as the air space around and above it within the crop canopy. The manifold consisted of 5 × 65 mm PVC pipes with ~45 × 6 mm holes spaced 20 mm apart drilled on the skyward facing surface of the pipes [34]. The manifold pipes were installed so an even amount of crop row was heated within each plot and between replicated heated plots. The sections of the manifold were secured with screws and sealed with self-amalgamating silicon and/or duct tape to prevent fittings from separating with air pressure and expansion and contraction caused by cyclic heating and cooling of the manifold. A heat-resistant flexible rubber hose (Flex-flow, Hoseco, Welshpool, WA, Australia), secured by hose clamps, was used to direct the hot air out and away from the heater inside the waterproof case to the manifold. The exhaust pipe was extended to prevent the heated plot or neighbouring plots from being “fertilised” by the CO_2_ produced by heating. An electronic fuel pump (that came with the heater) supplied winterised diesel (50:50 diesel:kerosene fuel mix) from a 5 L plastic diesel fuel container to the heater. The heater, fuel pump and 12 V DC digital thermostat temperature controller (STC-1000, unbranded eBay) with a PT-100 temperature probe were powered by a 12 V deep cycle battery (N70T, Century Yuasa Batteries, Forrestfield, WA, Australia). The battery was charged by a 160 W solar panel with an MPPT regulator (Kings, 4WD Supacentre, Bibra Lake, WA, Australia) facing north, ~30° vertical angle, mounted on six wooden stakes in the inter-plot gap between two neighbouring buffer plots, set above the growing crop canopy at the anticipated maturity height (800 mm AGL).

In 2018, a split-plot design was used to compare heated and non-heated treatments (Figure 1). The 1.7 × 5 m field plot was split in half with an insulated barrier between the sub-plots (Air-cell, Kingspan Insulation, Somerton, VIC, Australia). The subplots were randomly assigned to mitigate any effect of north- or south-facing treatments. A vertical array of unshielded thermocouples (T-Type, Temperature Controls Pty. Ltd., Sydney, NSW, Australia) at 200, 400, 600, 800 and 1000 mm AGL measured air temperature every minute in the centre of each treatment plot. The accuracy of the thermocouple is stated as 1 °C. Data were recorded by a data logger (DataTaker DT50-S3, Longtek, Glenbrook, NSW, Australia) paired with a calibrated weather station (CR5000, Campbell Scientific Australia Pty. Ltd., Garbutt, QLD, Australia) and sent to a server via the Telstra mobile network. 

The PT-100 temperature probe (accuracy stated at 1 °C) was calibrated at the start of the 2018 season with a Kestrel 5500 weather meter (temperature accuracy 0.5 °C, Kestrel Instruments, PA, USA). The plot heater was set to maximum heat output and would start heating when the air temperature at flag leaf height recorded by the PT-100 temperature probe fell to 2 °C. The 1.2 m^2^ area of the split plot was heated evenly by the manifold to 4 °C before the heater switched off. The heater would cycle on and off many times during a frost event. 

In 2019, further improvements were made to the design of the plot heater: (1) extending the manifold to 2 m in length to heat more crop row, enabling more plants to be measured and sampled; (2) the plot heater was switched on when the air temperature at 600 mm AGL (flag leaf height) fell to 2 °C, via a relay on the DT50 data logger (dataTaker DT50-S3, Lontek, NSW, Glenbrook, Australia), replacing the 12 V DC digital thermostat temperature controller; (3) relative humidity sensors were installed in addition to the thermocouples, replacing the thermocouples at 600 mm and 1000 mm in both treatments and; (4) having separate heated and non-heated plots (1.7 × 5 m each), rather than a split plot design as in 2018. Note: non-heated plots were adjacent length ways to heated plots in a paired plot design with an inter-plot gap of 500 mm between them; this allowed thermocouple wires to be laid on either side of this gap for temperature measurement. The weather station recording the temperature data, the waterproof case containing the heater, the fuel container and the solar panel were all located in this inter-plot gap in both 2018 and 2019 (Figure 2). 

Figure 2 shows the field installation and air temperature measurement systems used. In addition to this data, screen temperature and relative humidity were measured at the field site with a probe (Vaisala HMP60, ± 0.5 °C, Helsinki, Finland) housed within an enclosure (Campbell Scientific Model 413030-5A Sydney, NSW, Australia) at 1.2 m AGL. Wind speed and direction (Vaisala WMT50 Ultrasonic, Helsinki, Finland) were recorded as per Stutsel et al. (2019). 

A FLIR T420 (Teledyne FLIR, Wilsonville, OR, USA) was used to take thermal images of the plot heaters prior to dawn when in operation during frost events, to quantify the temperature of the manifold along its length and of the plants within the plot which it was heating. 

Plot heaters were installed prior to when the wheat plants reached Zadok growth stage Z39 [37]. The aim was for the heaters to be installed before the pollen young microspore stage as it was known to be frost sensitive [41]. In 2018, the three plot heaters and their weather stations were later removed (16 August 2018) at Z71 in time of sowing (ToS) block one (sown 12 April 2018) and moved to three new replicated plots in ToS block five (sown 10 May 2018) so that more data could be collected on additional field plots within that season. The plot heaters remained at their second location until Z87 when harvest index cuts were taken. In 2019, three additional plot heaters were built, so in total six heaters were deployed at the same developmental stages as 2018. Three were deployed in ToS block two (sown 17 April 2019) and three in ToS block six (sown 17 May 2019). This provided testing over two seasons × two different blocks or environments. 

### 2.3. In Season Measurements

**Figure 3:**
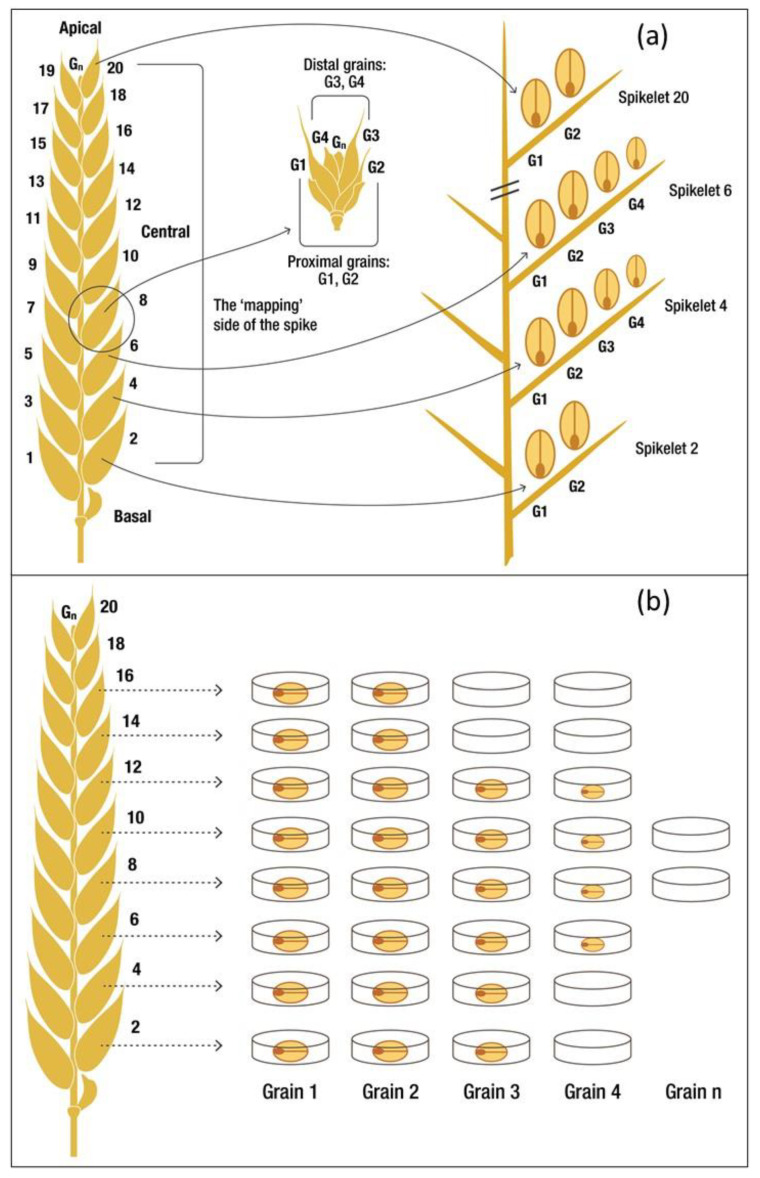
(**a**) A schematic diagram showing grain positions (G1, G2–proximal, G3 and G4–distal) at different spikelet positions. Spikelet 1 (S1) is the most basal, through spikelet position 20 (S20) is the most apical. (**b**) A schematic diagram showing the technique of grain mapping. Grains are individually removed from each spikelet and placed into their respective dish labelled with both the spikelet (Spikelet 2 to n) and floret (grain number from G1 to Gn).

The effectiveness of the plot heaters to protect wheat against frost damage was evaluated by assessments of sterility in proximal florets, grain mapping to determine the distribution of grains across the spike, yield and yield component traits. Zadok scores were collected weekly from flag leaf emergence (Z39) to the end of anthesis (Z69) [37]. Two development stages, 50% heading (Z55) and 50% anthesis dates (Z65) were estimated from these scores [42]. Floret sterility is the reduction in proximal (G1 and G2, see Figure 3a) grain number per spike expressed as a percentage of the total number of possible grains that could have formed in the proximal position within the floret (Equation (1)). When the spikes reached the medium milk stage (Z75), 30 primary tillers were collected and the floret sterility of the two proximal florets was determined (discarding the top and bottom florets) [21,40].

(1) $$Spike\;floret\;sterility=\left(\frac{sterile\;proximal\;florets}{total\;proximal\;florets}\right)\times 100$$

### 2.4. Grain Yield and Yield Component Measurements

At anthesis, 25 cm of crop row from each of the four inner plot rows was hand-cut above ground level using a quadrat (0.254 m^2^) [37]. These biomass cuts were dried at 70 °C for 48 h and weighed. Anthesis cuts were not taken in 2018 Heater+ plots as the removal of biomass would influence the canopy air movement in the plot and the amount of radiation entering the plot and soil surface during the day. At physiological maturity (Z87), 25 cm of crop row from each of the inner plot rows was hand-cut above ground level using a quadrat (0.254 m^2^). These harvest index (HI) cuts were oven dried at 70 °C for 36 h and weighed. Tillers were counted to determine spikes m^−2^, then carefully threshed to determine HI—the ratio of grain weight to above ground biomass [43,44]. Thirty main stem spikes per plot were sampled for floret sterility measurement during grain fill, four to six weeks after anthesis [21,45]. Note, HI for ToS 5 2018 could not be determined due to missing data. From the HI cuts, 15/20 primary spikes per replicated field plot in 2018/2019 were sub-sampled for grain mapping; see Figure 3 [46]. Mapped grains were dried at 60 °C for 48 h. The grain mapping method used was based on the work of Bremner and Rawson [47]; others have adapted this technique [48,49,50]. The method described in these past studies had the modification that undeveloped spikelets at the base of the spike were discarded and spikelet position one (S1) began at the first fully formed basal spikelet to (Sn) the most apical position, as in Figure 3a [51]. Additionally, grain set was mapped on one side of the spike [51,52] and mapped in lots of five spikes (three pseudo replicate subset groups × five spikes in 2018 and four pseudo replicate subset groups × five spikes in 2019) to decrease the grain weight measurement error by combining the grains from the same locations within the five spikes (e.g., combining all grains found in spike position 1 (basal), grain position 1 (proximal), with spike position 1 (basal), grain position 1 (proximal); see Figure 3b. Grain set data are presented as a proportion of grains present in each grain and spikelet position from the five spikes mapped × three pseudo replicates × three plot replicates. This was done so that the contribution of each grain position within the spikelet to grain set and grain weight could be determined, rather than just observing these traits across the whole spikelet [49]. Previously, we conducted a variance component analysis to determine the optimal sample size of spikes for grain mapping [51] using the approach of Snedecor and Cochran [53]. The optimal sample size of 15 and 20 spikes that was used in each respective season was based on the number of grain positions we had previously measured in Wyalkatchem at the same site in a field trial in 2017. Grain yield and yield components were determined from the whole plot (1.7 × 3 m, 1 m from each end of the plot was removed to eliminate edge effects) using a small plot harvester with settings optimised to retain the small frost-affected grains. 

Briefly, the yield components were determined as follows. Average grain weight (GW_a_) was determined from the harvested grain by counting 100 random grains and determining the total grain weight twice (G_r1_ + G_r2_), then averaging these weights (Equation (2)). If the variation between the two weights was >10%, then a third measurement of 100 random grains (G_r3_) was taken [44]. Grains m^−2^ (G_m_) was determined by dividing the total weight of grain threshed from the HI cut (G_HI_) by the average grain weight (GW_a_) (Equation (3)). Grains spike^−1^ was determined by dividing grains m^−2^ (G_m_) by spikes m^−2^ (S_m_) (Equation (4)).

(2) $${\mathrm{GW}}_{a}=\frac{G_{r1\;}+{\;G}_{r2\;}\;}{200}\;\mathrm{or}\;{\mathrm{GW}}_{a}=\frac{G_{r1\;}+{\;G}_{r2\;}+{\;G}_{r3}}{300}$$

(3) $$G_{m}=\frac{G_{\mathrm{HI}}\;}{{\mathrm{GW}}_{a}}$$

(4) $$G_{s}=\frac{G_{m}}{S_{m}}$$

The floret sterility, yield components and grain yield were analysed using a linear mixed model in the statiscial program Genstat^®^ 20th Edition, with the frost treatment (Heater+ or Heater−) as a fixed effect and plot replicate as a random term [54] and each sowing date was treated as a different environment and analysed separately. Predicted means are presented as *n* = 3. Significance tests were made using Fisher’s protected LSD test (*p* < 0.05) with each ToS compared separately when comparing treatments across measured traits as noted in the tables, with plot replicate as the grouping factor.

A linear mixed model was used to analyse grain set and grain weight within spikes; I.D. (grain and spike position information, e.g., G1S1, G2S2, etc., Figure 3) as a fixed term and plot replicate as a random term [54]. The interactions between individual grain weight and grain number were not included in the analysis as per Acreche and Slafer [49] and Feng et al. [55]. 

The relative contribution of grain at each position was calculated as per the formula reported in Feng et al. [55]. The grain weight from each grain position (Gn) was summed across all spikelets for each plot replicate and analysed using a linear mixed model with I.D. as a fixed term and plot replicate as a random term. The summed grain weight was divided by the total grain weight from all positions (G1 to G4) and expressed as a percentage.

## 3. Results

### 3.1. Frost Events 

**Figure 4:**
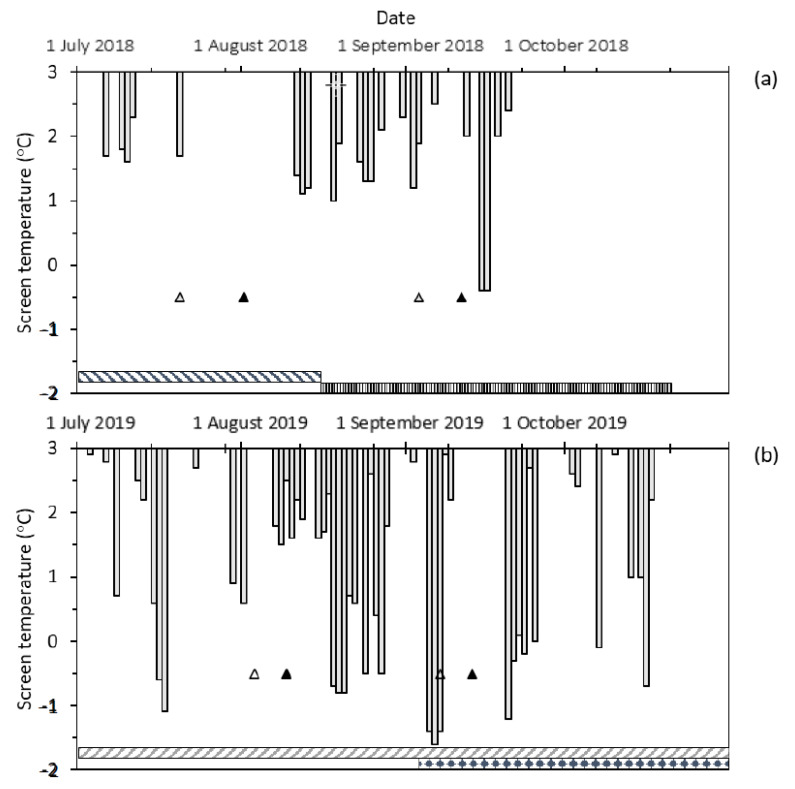
The timing of heading (Z55 open triangles) and anthesis dates (Z65 black triangles) for Wyalkatchem with frost events (screen temperatures ≤ 2 °C, 1200 mm above ground level) measured by an onsite weather station (Campbell Scientific) at Dale from July to October 2018 (**a**) and 2019 (**b**). The horizontal textured bars show the period during which the plot heaters were in place. Sowing dates for the corresponding heading and anthesis dates are: mid-April, 12 April 2018 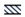 and early May, 10 May 2018 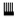 (**a**), mid-April, 17 April 2019 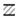 and mid-May, 17 May 2019 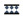 (**b**).

The frosts provided numerous opportunities to test the plot heater to provide non-frosted control plots when adjacent plots were at sub-zero degree temperatures on multiple occasions, using three different heaters in two environments (ToS blocks) per season. Frost events were frequent in 2018 and 2019 at Dale, with a cumulative total of four and six occurring throughout the heading and anthesis windows of Wyalkatchem over two sowing dates (Figure 4). The protection periods are noted in Figure 4 by shaded bars at the bottom of the graph. 

**Figure 5:**
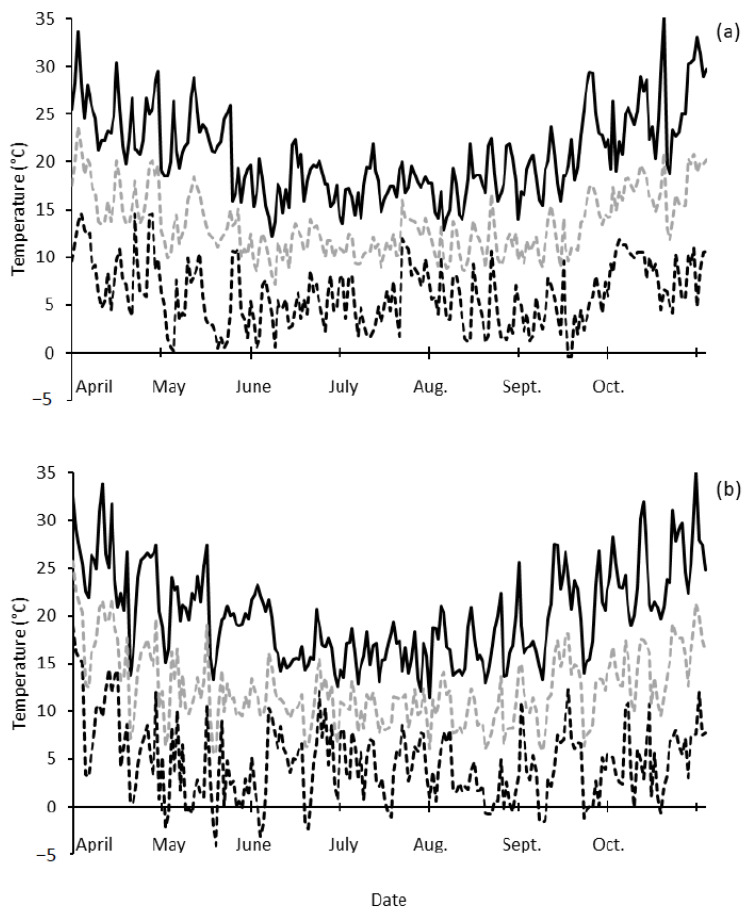
The daily march of minimum (dashed black line), maximum (solid black line) and average screen air temperature (dashed grey line) for the typical growing season in Western Australia from April to October 2018 (**a**) and 2019 (**b**) measured by an onsite weather station (Campbell Scientific) at Dale.

In 2018 and 2019 at the field site, there were 22 and 33 natural frost events (screen temperature ≤ 2 °C) from July to October. In 2018, there was one frost that occurred during Z55 (20 July 2018) and Z65 (1 August 2018) for mid-April sown Wyalkatchem; there were no other frosts within one week either side of these dates. For mid-May sown Wyalkatchem there were three frosts during the same development window (3 September 2018 to 11 September 2018); there were three frosts that occurred at the late anthesis stage within one week after Z65. More frost events occurred in 2019 with lower minimum temperatures and longer chilling duration (i.e., the cumulative duration < 5 °C screen temperature) compared to 2018. There were five frost events from 1 August 2019 to 10 August 2019 when Wyalkatchem sown on 17 April 2019 was at Z55 and Z65 (Figure 4). Up to 12 frosts occurred from one week before Z55, during Z55 to Z65, and one week after Z65 (30 July 2019 to 18 August 2019). For mid-May sown Wyalkatchem, there were two frosts that occurred between Z55 and Z65. Up to four occurred within one week before Z55, during Z55 to Z65, and one week after Z65 (2 September 2019 to 20 September 2019). Frost events in both seasons caused significant amounts of floret sterility in the spikes (20–60%). Figure 5 shows there was a lack of frost events and high temperatures at the end of the season. This resulted in low amounts of floret sterility with later sowing, in particular mid-May 2019 (see Section 3.3 “Yield and yield components of Wyalkatchem after frost in heated and non-heated plots”) and other later (June) sown plots of Wyalkatchem. The maximum temperature did not exceed 28 °C from July to September 2019 and only six days were above 25 °C during the anthesis period (Figure 5); at temperatures > 30 °C, some pollen sterility occurs due to heat rather than frost [56,57]. This provides evidence that the floret sterility present in spike samples was due to frost, and not another abiotic stress (heat or terminal drought).

### 3.2. Plot Heater Performance

**Figure 6:**
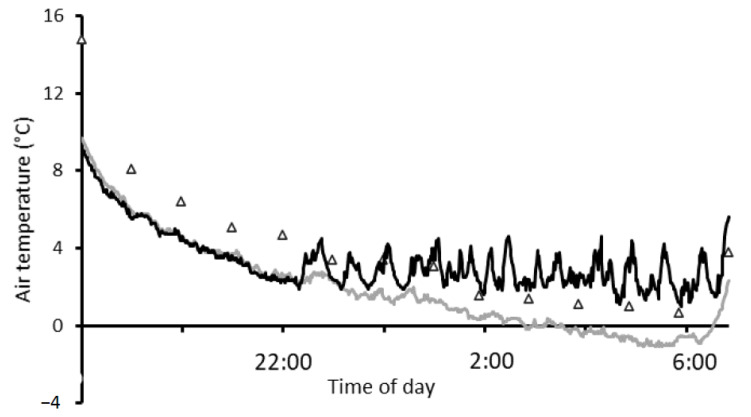
A typical frost event temperature profile depicting hourly (7 p.m. to 7 a.m.) screen air temperature (triangles—1200 mm above ground level—AGL from the onsite weather station) and minute canopy air temperature (an unshielded thermocouple 800 mm AGL T-type, Temperature Controls Pty. Ltd., Sydney, NSW, Australia) heated (black line) and non-heated (grey line) during heading of mid-May sown Wyalkatchem (Z55) on 7 September 2019.

Wheat plants experienced a large change in air temperature from daytime maximum to pre-dawn minimum during frost events. A typical frost event can be seen in Figure 6, hourly screen air temperature (from the onsite weather station) and minute canopy air temperature (from an unshielded thermocouple at 800 mm AGL) when mid-May sown Wyalkatchem was heading (Z55) on 7 September 2019. The temperature differences in these heated and non-heated plots were typical of frost events that occurred over the two seasons in 2018 and 2019. Screen temperatures were warmer than the unshielded canopy air temperature in non-heated plots by 1.3 to 1.8 °C from 12 a.m. to 7 a.m. (Figure 6). Over the 12 h time period, the screen temperature fluctuated 21.5 °C from the daytime maximum (22 °C) to the pre-dawn minimum (0.5 °C). 

**Figure 7:**
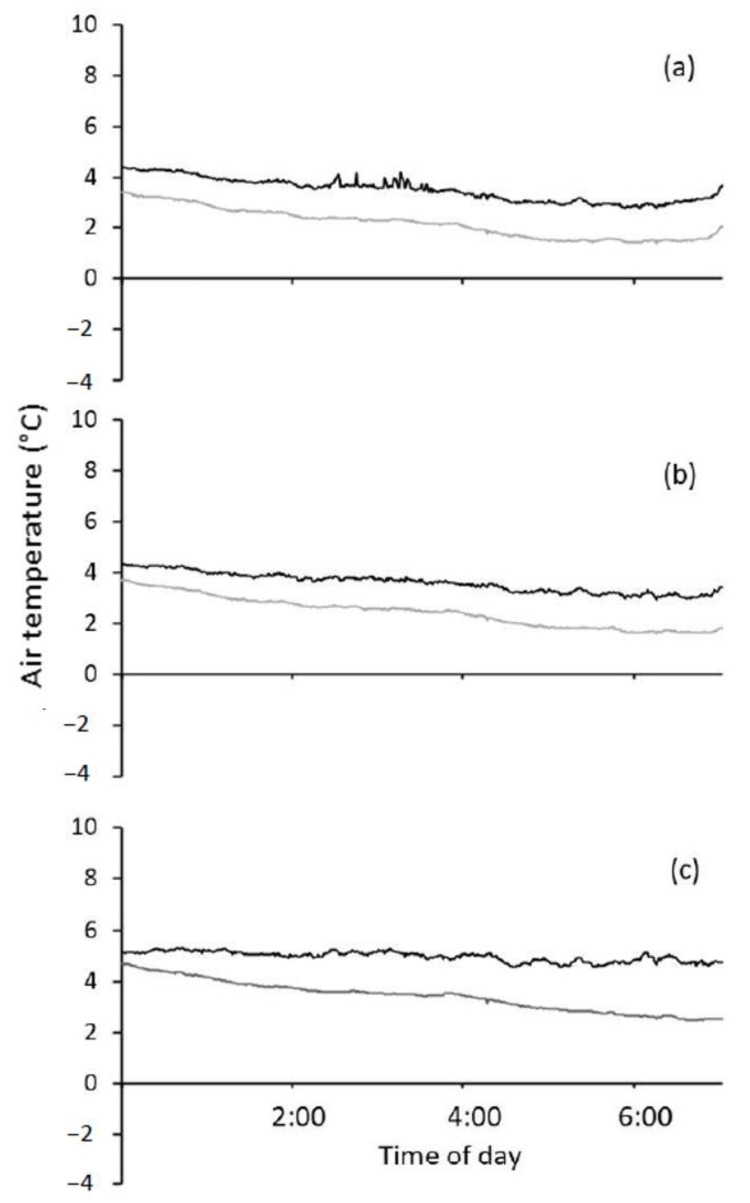
Average air temperature recorded in heated (black line) and adjacent unheated (grey line) plots (*n* = 3) at 1000 mm (**a**), 800 mm (**b**) and 400 mm (**c**) above ground level from frost events during July to October 2019. Air temperatures were measured by an unshielded thermocouple (T-type, Temperature Controls Pty. Ltd., Sydney, NSW, Australia) in mid-April-sown Wyalkatchem wheat plots.

The plot heaters were able to maintain air temperatures at canopy height above 0 °C (freezing) during frost events that occurred in 2018 and 2019 (Figure 7 and Supplementary Figure S1). The heated plots are maintained at 2–4 °C warmer than the non-heated plots whenever the air temperature at canopy height in the non-heated plot is <2 °C (Supplementary Figure S1). The durations of temperatures below 5 °C (where chilling and cold damage can result) were not different between heated plots and non-heated plots. For September 7th, frost durations of chilling temperatures overnight in heated plots were 678 (Heater 5) and 701 min (Heater 6) and in non-heated plots were 709 (adjacent Heater 5) and 728 min (adjacent Heater 6) (Heater 5 is shown in Figure 6). For the September 6th frost in Supplementary Figure S2, durations spent at chilling temperatures overnight in heated plots were 739 (Heater 5) and 758 min (Heater 6) and in non-heated plots were 729 (adjacent Heater 5) and 759 min (adjacent Heater 6). There was also good replication between the different heated plots to maintain canopy temperatures above freezing (Supplementary Figure S2). 

It was observed that there was a temperature differential from the origin of the plot heater manifold to the end of the manifold of ~40 °C (Supplementary Figure S3b) during a frost event on 18 August 2019 when the air temperature fell to −0.7 °C inside the screen (Figure 4). The extent of the heated area was contained within the plot (Supplementary Figure S3b). The surface temperature of some of the flag leaves is still below 0 °C (blue) in unheated areas around the edge of the plot. The non-heated plots were at −4 to −4.5 °C in the thermal image as per the outside of the plots shown there. Some flag leaves in the heated sections in Supplementary Figure S3b show surface temperatures approaching 0 °C (blue). These leaves were orientated horizontally and still had cooled dew on the adaxial surface which the rising warmer air from the heater had not warmed/evaporated. Visual leaf tissue damage occurred on horizontally orientated sections of flag leaves in non-heated plots, where cold dew pooled and presumably froze (Supplementary Figure S4a), but was not observed on these areas in heated plots. In summary, thermography demonstrated that the heater was effectively warming the plants within the plot without the heat spreading to neighbouring plots.

Visual field observations in 2019 and in predawn field thermography (Supplementary Figure S3b) during frost events indicated that heaters could protect spikes in addition to flag leaves. The coldest air temperatures measured by unshielded thermocouples were 800 mm above ground level (Supplementary Figure S2a,b). The top of the spikes was measured to be ~620 mm. Plant heights and the coldest vertical air height were consistent across frost events, ToS and seasons. Therefore, spikes would be located in air temperatures during a frost between those of (a), (b) and (c), (d) seen in Supplementary Figure S2. Air temperatures at the top of the canopy during the coldest frost (0.5 °C screen temperature) in September 2019 at some plots reached near 0 °C (Supplementary Figure S2d). However, for the rest of the frosts which were milder in minimum air temperature, the air temperature around spikes was kept well above 0 °C. 

**Figure 8:**
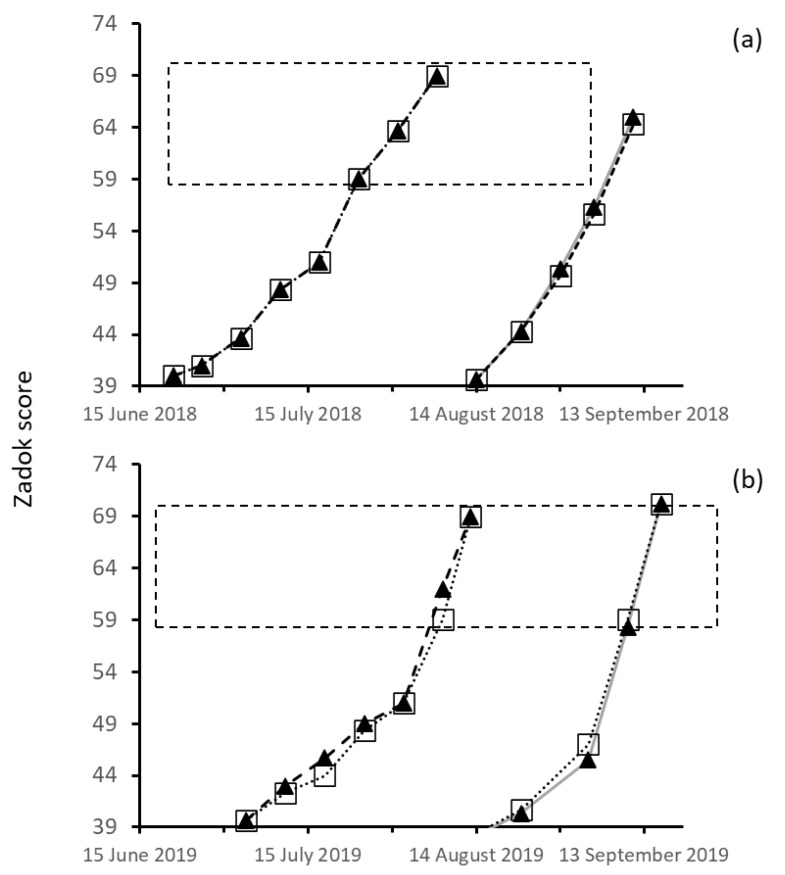
The crop development Zadok scores from Z40 to Z70 of heated (black triangle) and unheated (hollow square) plots (*n* = 3) in 2018 (**a**) and 2019 (**b**) seasons at Dale. Values are average Zadok plot scores (*n* = 3). Note: sub-plots were used in 2018 and paired plots were used in 2019 for heated and unheated treatments. The dashed box highlights the main susceptible period for wheat plants at the anthesis stage.

The plot heaters did not alter the phenology of the heated plot compared to the non-heated plots (Figure 8). Crop development was the same in the two different treatments in both the 2018 split-plot design and the paired plot design in 2019. Variation in the weekly scores was less than two points on the Zadok development scale (Figure 8).

### 3.3. Yield and Yield Components of Wyalkatchem after Frost in Heated and Non-Heated Plots

There was significant frost damage in the 2018 and mid-April-2019-sown Wyalkatchem with high floret sterility (30–60%) and low HI (0.06–0.23) (Table 1). Heated plots had 1.0 to 1.5 t ha^−1^ more grain yield than the unheated plots. This higher grain yield, or lower yield loss due to frost, was reflected in higher harvest index (an increase of 0.07 to 0.16 in heated plots), reduced floret sterility (11 to 31% less damage in heated plots), higher grains spike^−1^ (17 grains spike^−1^ more, mid-April 2019) and grains m^−2^ (2200 and 4400 more grains m^−2^ in mid-April 2018 and 2019). Average grain weight was greater in heated plots in 2018, but not in 2019. Spikes m^−2^ were not affected by the heaters (Table 1). For a frost in mid-April 2019 a 3-fold reduction in grain yield for Heater− compared to Heater+ was observed. There was no difference between the treatments in ToS 6. These contrasting ToS blocks were used for further detailed analyses of frost damage on grain set and grain weight within the spike (see Section 3.4. Grain Set and Grain Weight within Spikes of Wyalkatchem in Heated and Non-Heated Plots).

### 3.4. Grain Set and Grain Weight within Spikes of Wyalkatchem in Heated and Non-Heated Plots

**Figure 9:**
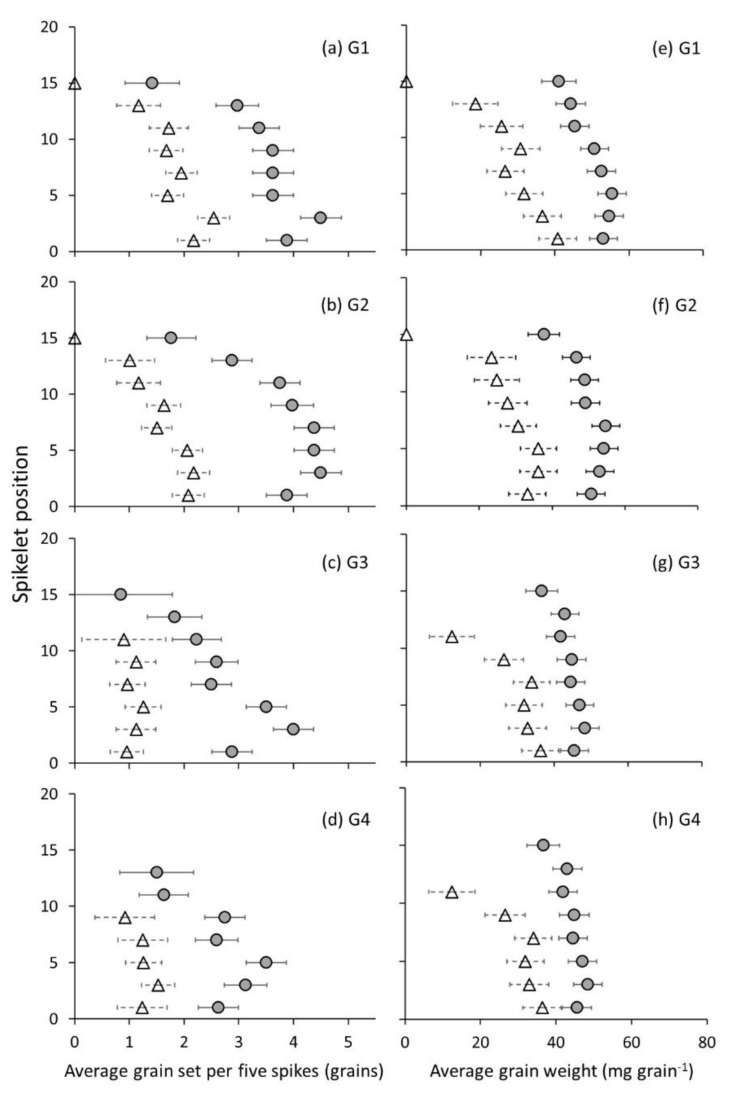
Values are predicted means (*n* = 45 spikes per data point) of average grain set and grain weight in heated and non-heated plots at a higher incidence of frost (mid-April 2019, 17 April 2019, Table 1 and Figure 4). Grain set is shown from G1 to G4 (**a**–**d**) (Figure 3) and average grain weight along the spike in each grain position of the spikelet is shown from G1 to G4 (**e**–**h**). Heated plot (grey circles) and non-heated plot (white triangles). Horizontal bars are ±standard errors.

**Figure 10:**
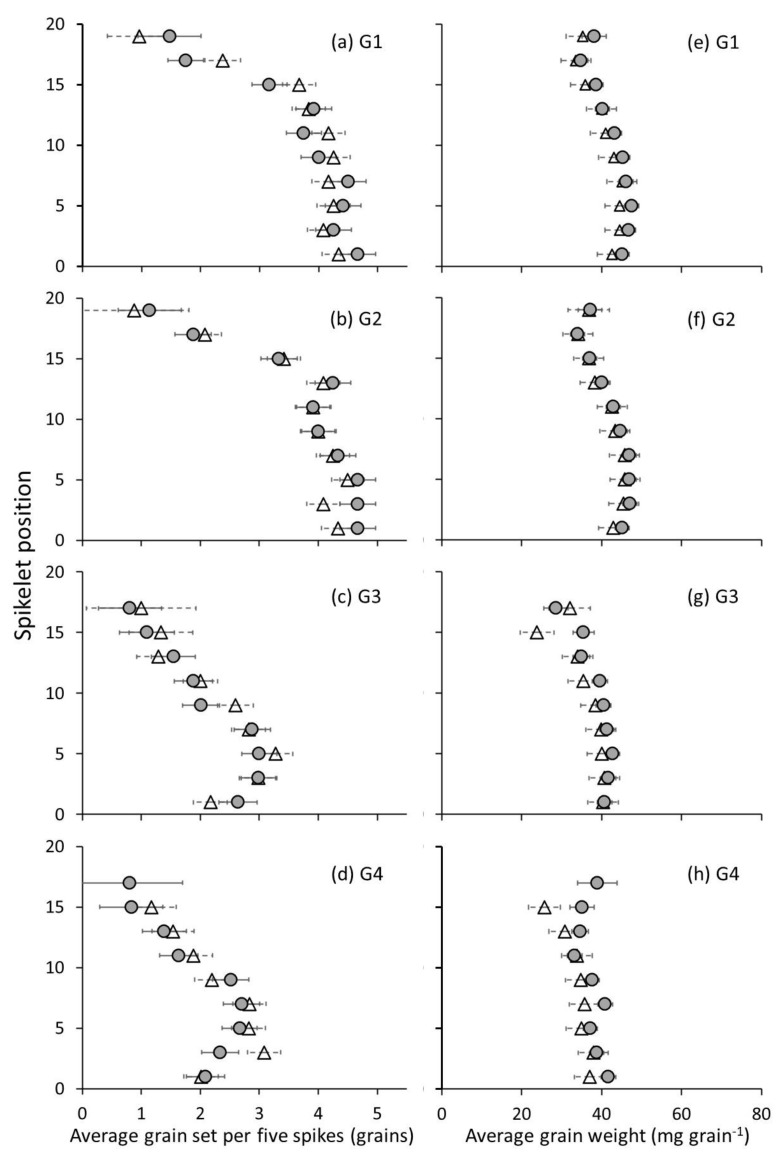
Values are predicted means (*n* = 45 spikes per data point) of average grain set and grain weight in heated and non-heated plots at a lower incidence of frost (mid-May 2019, 17 May 2019, Table 1, Figure 4). Grain set is shown from G1 to G4 (**a**–**d**) (Figure 3) and average grain weight along the spike in each grain position of the spikelet is shown from G1 to G4 (**e**–**h**). Heated plot (grey circles) and non-heated plot (white triangles). Horizontal bars are ± standard errors.

Apical spikelets are most affected by frost damage; at least one third of the apical proximal florets and one quarter of the distal florets had no grain set in them in both 2018 and 2019 (Figure 9 and Figure 10).

The differences in grain set and grain weight between the heated and non-heated plots were greatest in the proximal florets. Proximal florets also had the heaviest grains present at 50–60 mg grain^−1^ in heated plots and 20–40 mg grain^−1^ in non-heated plots under conditions of a higher incidence of frost (Figure 4 and Figure 9e,f). Grain set was approximately two-fold greater in heated versus non-heated plots in the proximal florets (Figure 9a,b) in April sown Wyalkatchem, 2019. 

Under a lower incidence of frost (Figure 4), distal florets (G3 and G4) were significant contributors to grain number (Figure 10c,d), grain weight per spike (Figure 10g,h) and grain yield. 

Central spikelets were the most responsive to frost damage. In mid-April 2019, under the high incidence of frost (Figure 4), the central spikelets in non-heated plots had an average grain set of 1.5–2 grains per spikelet in proximal florets; heated plots, by contrast, on average had more than double that, with 3.5–4.5 grains per spikelet. In mid-May 2019, under low frost (Figure 4), grain set was 4–5 grains per spikelet in proximal florets and 2.5–3 grains in distal florets with no difference with the heaters. Grain weight was also stable for both treatments at 40–45 mg grain^−1^ at all central spikelet positions (Figure 10e–h). 

### 3.5. The Relative Contribution of Grain Position to Spike Grain Weight and Grain Number per Spike

The contribution of grain position to spike weight and ultimately grain yield was most for G1 and G2, followed by G3 and then G4 (Figure 9 and Figure 10). Under low incidence of frost (<10% floret sterility) at mid-May in 2019 (Figure 4), the heated and non-heated treatments proximal grain positions G1 and G2 contributed over 36–37% of the spike weight and ultimately grain yield, followed by G3 (15%), then G4 (13 and 12%) (Figure 10b). However, when spikes were damaged by frost and floret sterility was present in higher amounts (30–60%), patterns of grain set were altered. In 2019, in mid-April heated plots, G1 and G2 contributed 31 and 35% of the spike weight, G3 19% and G4 15% (Figure 10a). In adjacent non-heated plots in mid-April with a high incidence of frost, the contributions to spike weight were: G1 38%, G2 37%, G3 13% and G4 12% (Figure 10a). There was a greater percentage reduction in the proportional contribution to the spike weight of G3 and G4 in the non-heated plots compared to G1 and G2. Despite this change, the total grain weights in each grain position revealed that in non-heated plots there was a significant reduction of all grain positions: G1 weights were reduced by 70%, G2 by 74%, G3 18% and G4 20% (Figure 10c). Therefore, the remaining grains in G1 and G2 were of considerable weight (25–40 mg grain^−1^) and of enough number to still maintain a contribution of 38 and 37% to grain yield for frost-damaged non-heated plots (Figure 10a). 

**Figure 11:**
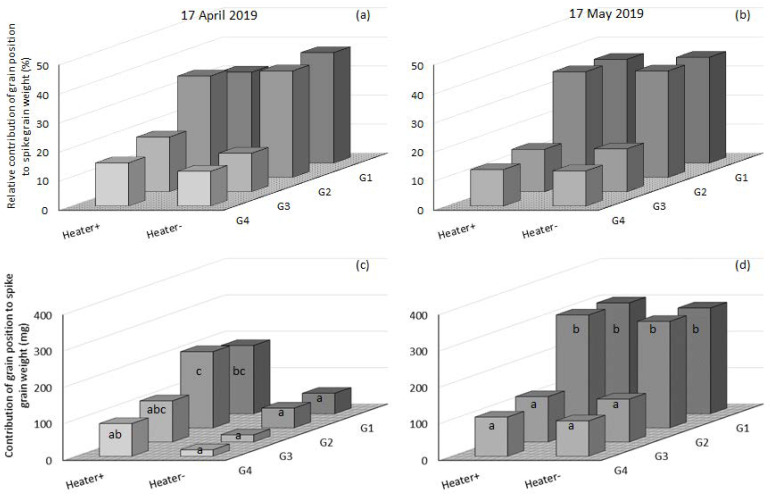
The relative contribution of grain at each position of the spikelet (grain position) to spike grain weight in Wyalkatchem under incidence of frost sown in mid-April (17 April 2019 (**a**)) and low incidence of frost mid-May (17 May 2019 (**b**)) sampled from heated and non-heated plots. Absolute spike grain weights for the same sowing dates are shown on (**c**,**d**) with significance (*p* < 0.05) noted by different lowercase letters for each sowing date compared separately. Relative contributions are calculated from the predicted means of absolute spike grain weights (*n* = 45 spikes per data column).

In 2018, contrasting amounts of floret sterility were measured in heated and non-heated plots for mid-April (33 and 44%) and mid-May (29 and 59%) (Table 1). In mid-April, proximal grains G1 and G2 in heated plots contributed 35 and 38% of the spike weight, followed by G3 18% and G4 9%; in the frosted non-heated plots, the contributions were G1 36%, G2 40%, G3 14% and G4 10% (Figure 11a). The reduction for each position in total grain weight from non-heated compared to the heated treatment was: G1 23%, G2 21%, G3 57% and G4 23% (Figure 11c). So, just as in mid-April in 2019, 2018 saw the proportional contribution of G1 and G2 increase compared to G3 and G4 in non-heated plots. However, the reduction in grain weight of each position per spike was different: G1 and G2 weights were reduced by 47 and 53% more in mid-April in 2019, whereas G3 and G4 had a 40% and 3% greater reduction in mid-April 2018 than mid-April in 2019. In later-sown mid-May in 2018, Wyalkatchem proximal grains G1 and G2 in heated plots contributed 40 and 37%, G3 15% and G4 8%; in frosted non-heated plots, the contributions were G1 32%, G2 37%, G3 23% and G4 8% (Figure 11b). In this frost environment, an 8% increase in the relative contribution of G3 was observed; in contrast to the two other frosted environments mentioned above, there was a decrease in the relative contribution of G1, while G2 and G4 did not change compared to the heated plots (Figure 11b). G1 and G2 had a 42% and 22% decrease in spike grain weight, G3 a 13% increase and G4 a 27% decrease between heated and frosted non-heated treatments (Figure 11d).

**Figure 12:**
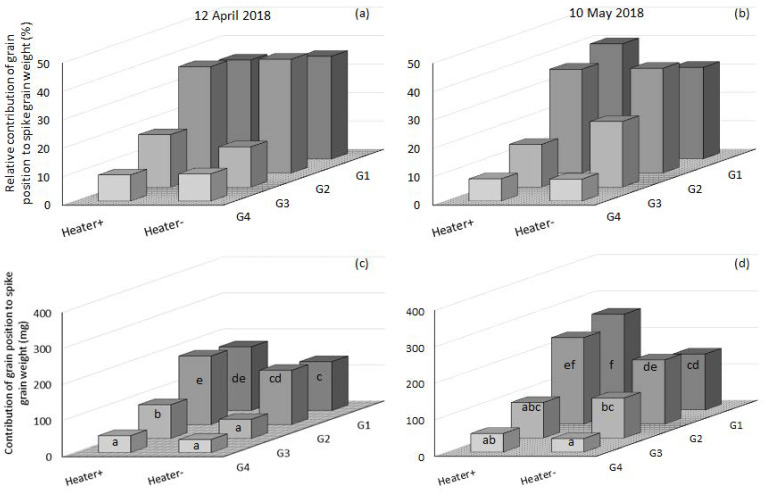
The relative contribution of grain at each position of the spikelet (grain position) to spike grain weight in Wyalkatchem sown in mid-April (12 April 2018 (**a**)) and mid-May (10 May 2018 (**b**)) sampled from heated and frosted non-heated plots. Absolute spike grain weights for the same sowing dates are shown on (**c**,**d**) with significance (*p* < 0.05) noted by letters with each sowing date compared separately. Relative contributions are calculated from the predicted means of absolute spike grain weights (*n* = 45 spikes per data column).

When frost damage sterilises proximal florets, distal florets can compensate by increasing their grain number and weight (Figure 12b G3). The weight of distal grains in the central and basal florets in non-heated plots was still 30–35 mg grain^−1^ and was not very difference from the weight in heated plots. Whereas in the proximal florets there was a much greater difference between heated and non-heated plots, both in terms of grain set and grain weight. 

In conclusion, between mid-April 2018 and 2019 we measured a proportional contribution of G1 and G2 increase compared to G3 and G4 when comparing frosted non-heated plots to their adjacent heated plots. In a more severe frost damage environment, mid-May 2018 (Heater+ 30% and Heater− 60% floret sterility, Table 1), the proportional contribution of G1 decreased and G3 increased, showing some indication of compensation occurring when the dominant proximal grain positions were sterilised. The extent of the changes in actual weights and grain numbers varied with frost exposure and timing across environments.

## 4. Discussion

Plot heaters were able to reduce the severity and duration of frosts and resultant frost damage to wheat plants compared to naturally frosted non-heated plots in all frost environments experienced (Table 1). For these frost events, the heaters were able to sufficiently heat the plants to prevent freezing frost damage during the critical, flag leaf extension, heading and anthesis stages (see Figure 6 as an example for the coldest frost in 2019). Heaters increased the canopy air temperature by 0.5–1 °C at the coldest part of the canopy (800 mm) and the coldest time of the frost at or just prior to dawn in this example. The protection from more severe frost damage by the heaters was reflected in the floret sterility data from heated plots being significantly lower across all environments compared to non-heated plots. Floret sterility was reduced by 10 to 30% across the environments in frost-damaged sowing windows. On average, grain number was 2200 to 4400 grains m^−2^ greater in heated plots compared to frosted non-heated ones. This translated to a 1.05 to 1.60 t ha^−1^ improvement in grain yield. Therefore, the grain yield lost to freezing frost damage was 1.05 to 1.60 t ha^−1^. Plot heaters were successful in heating an enlarged area of field plot without the heat escaping into the neighbouring plots, and as more frosts occur on still nights with very low wind speed (<1.0 m s^−1^), the hot air simply rose, warming the canopy above. Thermal images confirmed that no radiant heat was being experienced by neighbouring plots, as reported also in Stutsel et al. [34]. The heater design meant that heating the crop canopy occurred from the base of the plant upwards. This would have assisted in preventing the freezing of plant tissue that occurs in the field from the ground up [19]. In conclusion, plot heaters were able to maintain canopy air temperatures at or above 2 °C when the ambient air temperature was at or below 0 °C for frost events in 2018 and 2019. Plot heaters are useful tools in moderating the effects of frost on wheat plants in randomised field trials to enable the further study of the effect of frost on different stages of wheat development under field conditions.

In the experiments, the heater was found to effectively warm the plots, leading to a reduction in frost damage (floret sterility), resulting in improvements in yield and yield components. Thermography was able to confirm that the heaters prevented freezing damage from occurring. However, despite this result, there was still floret sterility (9–33%) in heated plots in both 2018 and 2019 (Table 1). While temperatures did not reach freezing (≤0 °C), the Figure 6 example shows that temperatures did enter the chilling stress temperature range (0–5 °C) [58]. Chilling temperatures were reached in frost events noted in Figure 4 in both heated and non-heated plots in 2018 and 2019 (Figure 7 and Supplementary Figure S1). This suggests that the spikes were still within the coldest vertical gradient of temperature, despite the top of the leaf canopy being warmed to 1–2 °C when the non-heated plots were at temperatures below 0 °C (Figure 6). Consequently, spikes were still exposed to chilling temperatures. 

Despite the reduction in frost freezing damage, the heated plots would contain chilling-damaged plants and the frosted non-heated plots containing chilling- + freezing-damaged plants. The resultant (chilling) stress produced 21 to 33% floret sterility in heated treatments (Table 1). The reason for floret sterility to be present in heated plots is likely due to chilling damage resulting from plants exposed to air temperatures <5 °C. Chilling has been shown to be damaging to wheat plants at susceptible stages during jointing, booting/young microspore and anthesis [14,15,16,58]. Cheong et al. [41] (Supplementary Figure S1d) suggests the sensitivity is due to the developing pollen and its subsequent viability and ability to fertilise the ovule declined with increasing duration of chilling. A negative effect of chilling on pollen has been observed in other crops, such as rice and pulses [14,16,59]. The 21–33% of floret sterility is likely to be due to the combined effects of any young microspore chilling damage and pollen sterility, due to chilling temperatures at the post-heading stage which was still present in heated plots. Some of the sterility present in mid-April in 2018 heated plots was also likely due to grain frost damage that occurred after the heaters were shifted to mid-May sowing date at Z71 (Figure 4) and floret sterility measured in spikes at Z85 (harvest index cut grain samples confirmed the presence of grain frost damage (Supplementary Figure S5) [60,61]. In summary, the plot heaters can prevent freezing damage in frosts and enable the further study of the effect of freezing and chilling frost damage on different stages of wheat development under field conditions.

Measurements of grain set within the spike revealed that apical spikelets are most affected by frost damage. This result is consistent with other abiotic stresses such as heat, in which exposed spikes show symptoms of “tipping”: apical spikelets become bleached and are aborted [62]. Under controlled environment conditions, the apical spikelets of wheat were the most impacted by low temperature in wheat at jointing and booting stages [15]. Marcellos and Single [63] reported that the top of the canopy is the coldest point in a plant canopy during a frost. Therefore, it is reasonable to expect the greatest amount of sterility to occur in the apical spikelets and that the grain set data and the average grain weights along the spike would be impacted the most there. However, if FASE reduces the amount of assimilates reaching the spike containing the developing grains or reproductive organs, then two things are expected to occur: (a) the apical part of the spike will be affected first with the onset of stress (i.e., more sensitive), (b) the limitation on assimilates reaching the spike will cause the plant to preferentially direct resources to central and basal floret positions in the spike. Selection of wheat cultivars with lower apical spikelet sterility when exposed to post heading frosts would reduce the yield losses in the frost-prone wheat-growing regions of the world. 

The central spikelets were most responsive to changes in grain set and grain weight. This was also the region where there are more grain sets per spikelet in the heated treatment. Across the four environments, central spikelets set a higher number of grains per spike compared to apical and basal spikelets. In mid-April 2019 sown Wyalkatchem (which reached anthesis at a period of higher incidence of frost), the central spikelets in frosted non-heated plots had grain set of 1.5–2 grains per spikelet in proximal florets; while the heated plots had 3.5–4.5 grains per spikelet in proximal florets (Figure 8a,b). This represented the greatest difference in grain set between the treatments out of apical, central and basal regions of the spike. In mid-May 2019 sown Wyalkatchem, which reached anthesis at a period of lower incidence of frost, grain set was 4–5 grains per spikelet in proximal florets and 2.5–3 grains in distal florets (Figure 9a–d). Under this low floret sterility scenario, fruiting efficiency was high in this part of the spike and grain weight was stable along the spike at 40–45 mg grain^−1^. Slafer and Savin [64] found that the grain weight of central spikelets was more responsive to de-graining when they manipulated the source-sink relationship of two cultivars in a field experiment. Grain number in these spikelets appears to be more plastic and therefore represents an opportunity for selective breeding for higher fruiting efficiency in this part of the spike.

The dominance of grain positions G1 and G2 for spike weight and grain yield under varied frost damage severity was evident in our results. In conditions where spike floret sterility was 36, 44 and 59%, G1 and G2 still made up 38, 36 and 32% (G1) and 37, 40 and 37% (G2) of the total spike weight (Table 1 and Figure 9a,b and Figure 10a). However, the extent of dominance of G1 and G2 was reduced by frost damage (Figure 10c and Figure 11d). Other studies also found this dominance of the proximal grain positions and observed changes in grain weight with de-graining and changes through breeding [47,49,55]. There have been only two other studies that have mapped the grain in frost-damaged spikes known to the authors [52,65]. The former of these studies reported grain set within the spikelet, contrasting six different cultivars. In support of what we found, grain set varied with sowing time and frost exposure. The novelty of this new research is that grain set changes on a per-floret basis have been reported, with a frost control treatment (i.e., a plot heater). By using a control for the frost treatment, we have demonstrated that the apical part of the spike is most affected by frost damage, G1 and G2 contribute the most to grain yield, and when grain set in G1 and G2 is reduced, grain yield is significantly reduced. 

Distal florets (G3 and G4) were still significant contributors to grain number per spike, grain weight and grain yield in the presence of frost damage (Figure 11d, G3, Heater−). In situations where proximal florets (G1 and G2) were sterilised due to frost damage, these distal florets then become even more important to maintaining grain number and grain weight within the spike, and ultimately grain yield. Wheat breeding over the decades has influenced the contribution of grain positions with the spikelet to grain yield. Acreche and Slafer [49] found a traditional CIMMYT cultivar with higher average grain weight and lower grain number, and that had higher proximal and lower distal grain numbers in the central spikelets. Compared to a modern French cultivar with lower average grain weight and higher grain number, it had lower proximal and higher distal grain numbers in the central spikelets. In China, wheat breeding since the 1950s has seen an increase in the contribution of grain number from G3 and G4; the relative contributions of G4 have increased over time [55]. The introduction of *Rht* genes to increase harvest index and grain yield has increased the contributions of distal grains to grain number and grain weight [66]. In one out of the four ToS × year environments (mid-April in 2018) in our experiments, we observed that the average grain weight declined as grain m^−2^ declined (Table 1). Some of this could have been due to grain frost damage (Supplementary Figure S5). It is generally accepted that average grain weight declines as grains m^−2^ increases [49,67]. Therefore, it appears that frost damage can have the inverse effect on these yield components to what is commonly observed. This would be conditional on the timing and severity of frost events, which is variable at best. Part of the explanation could be that in reducing grain set in proximal florets, the proportional contribution and weight of typically smaller grains (G3 and G4) increase from distal florets [49]. This can be observed in the reduction in the proportion on grain set in G3 mid-May in 2018 (Figure 11b,d). However, the same observation did not occur in April 2018 or mid-April 2019. In frost-prone environments, selective breeding to increase the contribution of distal grains to grain number and average grain weight could provide a greater buffer against the loss of grain numbers caused by post-heading frost damage. 

Evidence of compensation in commercial frosted crops through increases in grain weight and re-tillering is often observed in the field. We found evidence of compensation through increases in grain weight as seen in G3 in 2018 mid-May sown Wyalkatchem (Figure 11b). However, this same result did not occur in G3 for mid-April sown Wyalkatchem in 2018 and 2019, or in mid-May sown Wyalkatchem in 2019 (Figure 9a,b and Figure 11a). Calderini and Reynolds [48] results using synthetic hexaploid wheat showed that the timing of the de-graining had a significant effect on the grain weight change. De-graining at heading significantly increased the weight of remaining grains. However, when de-graining occurred after anthesis, there was no increase in final grain weight. It is likely that similar compensation by increases in grain weight will only occur if frost(s), resultant sterility and reductions in grain number occur(s) at heading, prior to anthesis. However, there will be limits to the amount of the compensation that can occur through increases in grain weight, carpel weight being a primary factor [48,68,69]. As grain number is the main driver of grain yield [49,64,70,71], we believe there would be a tipping point at which grain weight cannot compensate for the loss of grain number (Table 1). In mid-April 2019 sown Wyalkatchem, such a tipping point was exceeded with grain number being reduced by frost damage to one quarter that of the heated control. Comparatively, mid-April 2018 sown Wyalkatchem did not reach such a tipping point; the grain number, although reduced, did not plummet and the result was the grain yield reduction was not as severe. In both these examples, grain weight was still higher in the heated control, although the difference was significant for 2018, not 2019. In conclusion, compensation through grain weight increase was possible in frost-affected spikes but is dependent on: (a) the timing, duration and number of frost events; (b) the number of sterile florets; and (c) carpel size of fertile florets.

Seasonal timing, intensity and severity of frost events are variable and hence the effect that frost will have on grain set and grain weight within spikes of wheat will be variable. The variability in floret sterility and grain weight contribution of each grain position to spike weight between seasons can likely be explained by the differences in the number and timing of frost events and when it coincidences with more susceptible growth stages (Figure 4) [12,52,72,73]. However, the variation within each season, sowing date and plot, could also be due to other factors that relate to the freezing process, the presence of ice nucleators and supercooling [17,74,75,76]. Livingston et al. [19] demonstrated in the field that wheat plants freeze one at a time from the ground up, in a random manner. The older leaves freeze first, followed by the younger and then the stems. In-field observations via thermography did confirm this ground-up freezing process during spring frosts in Australian field conditions [77]. The temperature data from this study, field observations (thermography) and the lack of stem/crown frost damage symptoms demonstrate that ground-up freezing was prevented by the plot heaters (Figure 5, Figure 6 and Figure 7). However cold and chilling damage from the low temperatures (0–8 °C) above the canopy was still present (see Figure 4, Figure 7 and Supplementary Figure S1). Therefore, the plot heaters are useful to study the effects of chilling and cold damage on developing spikes, their grain set and size, while preventing freezing damage. Further research could examine the ratio of chilling and cold damage versus freezing damage by designing an experiment with multiple heaters and using them in such a way as to have several frost “doses” to create a frost dose-response curve. Additionally, different stages of development could be protected to varying extents to develop dose and timing response curves. This data would be useful in developing better frost damage functions and overcoming the limitations of frost covers which can shade plants and still have <0 °C temperatures present in covered plots [4,33,78]. Models with an improved frost damage function based on data and not assumptions [26,79] can then be used to more accurately model future climate scenarios and more accurately determine the economic cost of frost damage in Australia [24,27,28,29,30,79] and optimal agronomic management decisions [23,31]. The plot heaters offer potential economic benefits to the Australian grain industry, assisting screening of wheat for reduced susceptibility to frost damage at the post-spike-emergence stage. Future collaborative research should aim to combine frost damage modelling and economic approaches, with the improved understanding of frost damage gained by having a frost “control”. 

## 5. Conclusions

Plot heaters are useful tools in moderating the effects of chilling (0–5 °C) and freezing (≤0 °C) temperatures during frost(s) (screen temperatures ≤ 2 °C) on wheat plants to enable the further study of the effect of frost on grain yield and its components. The present work evaluated responses of one wheat cultivar, Wyalkatchem, but we hypothesise that cultivars with a high concentration of grains in proximal florets (G1 and G2) are sensitive to yield loss when these grain positions are sterilised by frost as reductions in grain yield are attributed to decreased grain number per spike. Increasing the contribution of distal florets (G3 and G4) to grain number and grain weight could help maintain grain yield when sterility occurs in proximal florets. Central spikelets show greater plasticity to changes in grain number and weight than apical and basal spikelets under varying amounts of frost-induced sterility. Exploring the variation in commercial cultivars for this trait would improve our understanding of differences in cultivar performance under spring frosts in the field. Apical positions are particularly sensitive to cold stress. Future research should focus on understanding why this occurs and, if confirmed, breeding should aim to select cultivars with higher fertility in this part of the spike.

## Figures and Tables

**Table 1:** Values are predicted means (*n* = 3) of grain yield, floret sterility and yield components of wheat (*cv.* Wyalkatchem) in heated and non-heated plots for the 2018 and 2019 seasons at Dale, Western Australia. Lowercase superscript letters indicate significant mean differences tested using a Fisher’s protected LSD test (*p* < 0.05) for comparisons within each sowing date (ns = not significant). Data are taken from harvest grain samples ^A^ and the whole harvest index cut ^B^, anthesis biomass cut ^C^ (Z65) and floret sterility ^D^ from main stem spikes collected at Z75.

Sowing Date	Treatment	Grain Yield ^A^ (t ha^−1^)	Harvest Index ^B^ (%)	Anthesis Biomass ^C^ (t ha^−1^)	Floret Sterility ^D^ (%)	Grains ^B^ Spike^−1^	Grains ^B^ m^−2^ (000s)	Average Grain Weight ^A^ (mg grain^−1^)	Spikes ^B^ m^−2^
Mid-April 2018	Heater+	3.4 ^a^	0.23 ^a^	-	33 ^a^	-	9.5 ^a^	36.4 ^a^	-
	Heater−	2.4 ^b^	0.17 ^b^	7.1	44 ^b^	14	7.3 ^b^	33.0 ^b^	522
Mid-May 2018	Heater+	4.9 ^ns^	-	-	28 ^a^	-	9.9 ^ns^	49.4 ^a^	-
	Heater−	4.4 ^ns^	0.2	9.9	59 ^b^	18	8.5 ^ns^	51.7 ^b^	463
Mid-April 2019	Heater+	2.3 ^a^	0.22 ^a^	-	21 ^a^	25 ^a^	5.8 ^a^	48.3 ^a^	232 ^a^
	Heater−	0.8 ^b^	0.06 ^b^	7.2	36 ^b^	8 ^b^	1.4 ^b^	45.3 ^a^	209 ^a^
Mid-May 2019	Heater+	5.0 ^a^	0.40 ^a^	-	8 ^a^	41 ^a^	11.3 ^a^	44.3 ^a^	278 ^a^
	Heater−	4.5 ^a^	0.40 ^a^	9.	11.2 ^b^	40 ^a^	11.7 ^a^	43.2 ^a^	299 ^a^

## Data Availability

The data presented in this study are available on request from the corresponding author.

## Supplementary Materials

The following supporting information can be downloaded at: https://www.mdpi.com/2073-4425/13/4/578/s1, Figure S1: Average minute air temperature at 1000 (a), 800 (b), 600 (c), 400 (d) and 200 mm (e) above ground level (AGL) for the Heated plots (solid line) and adjacent Non-heated plots (grey line) for frost events in July to October 2018; Figure S2: Air temperature at 800 (a and b), 400 (c and d) and 200 mm (e and f) AGL for the Heated plot (solid line) and adjacent non-heated plot (grey line) on 6 September 2019. Data is from Heater 5 and Non-heated plot 5 (a, c, and e) and Heater 6 and non-heated plot 6 (b, d, and f); Figure S3: The diesel heater heating a field plot during a frost event at Dale August 18th, 2019 (a) digital photograph, (b) thermal image. In (a) the heated area can be seen by the darker canopy (inside dashed line box) which indicated that the heat kept most of the frost (white dew on the awns and spikes seen in the background) off the plants, i.e. prevented symptoms of plant frost exposure. In (b) the thermal image shows the warm PVC pipe and the colder canopy around the edge of the heated plot.; Figure S4: (a) Freezing damage to wheat flag leaves at Dale 6 September 2019. (b) The bleaching and plant tissue damage is isolated to sections of the flag leaves where they were horizontally orientated and cold dew pooled and froze there. Freezing damage presents itself bronze patches on leaves a few hours after the frost, this remains on the leaves for ~1–3 days; Figure S5: Images of grain samples from unheated plots harvest cuts ToS 1 (a) and ToS 5 (b) 2018. (a) shows clear evidence of grain damage due to frosts occurring at the grain filling stage (Zadoks growth stage Z70–77 from 7 to 14 August 2018) (Figure 3a and Figure 6a). This resulted in pinched and shrivelled grain like that seen in Cromey et al. 1998. (b) shows large grain size in unheated plots, despite moderate levels of flowering frost damage at (60% in heated) (Table 1). There were no frost events during grain filling stage (Figure 3a).
