# Correction: Leske, B.A.; Biddulph, T.B. Estimating Effects of Radiation Frost on Wheat Using a Field-Based Frost Control Treatment to Stop Freezing Damage. *Genes* 2022, *13*, 578

**DOI:** 10.3390/genes14030728

**Published:** 2023-03-16

**Authors:** Brenton A. Leske, Thomas Ben Biddulph

**Affiliations:** 1 The Department of Primary Industries and Regional Development, 3 Baron Hay Court, South Perth, WA 6151, Australia; brenton.leske@dpird.wa.gov.au; 2 The School of Agriculture and Environment, The University of Western Australia, 35 Stirling Highway, Crawley, WA 6009, Australia

## Error in Figure

In the original publication [1], there was a mistake in Figure 11 and Figure 12 as published. An error was discovered in the calculation of the values displayed in the figures which meant the values were of a lower magnitude than the correct value. The corrected Figure 11 and Figure 12 appear below. The authors state that the scientific conclusions are unaffected. This correction was approved by the Academic Editor. The original publication has also been updated.

## Figures and Tables

**Figure 11:**
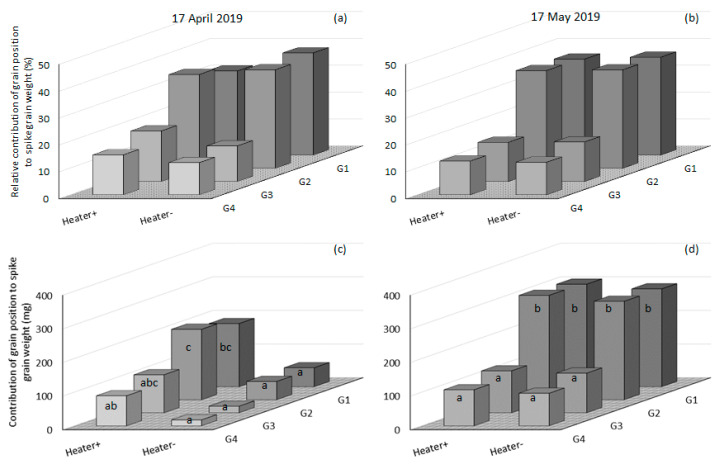
The relative contribution of grain at each position of the spikelet (grain position) to spike grain weight in Wyalkatchem under incidence of frost sown in mid-April (17 April 2019 (**a**)) and low incidence of frost mid-May (17 May 2019 (**b**)) sampled from heated and non-heated plots. Absolute spike grain weights for the same sowing dates are shown on (**c**,**d**) with significance (*p* < 0.05) noted by different lowercase letters for each sowing date compared separately. Relative contributions are calculated from the predicted means of absolute spike grain weights (n = 45 spikes per data column).

**Figure 12:**
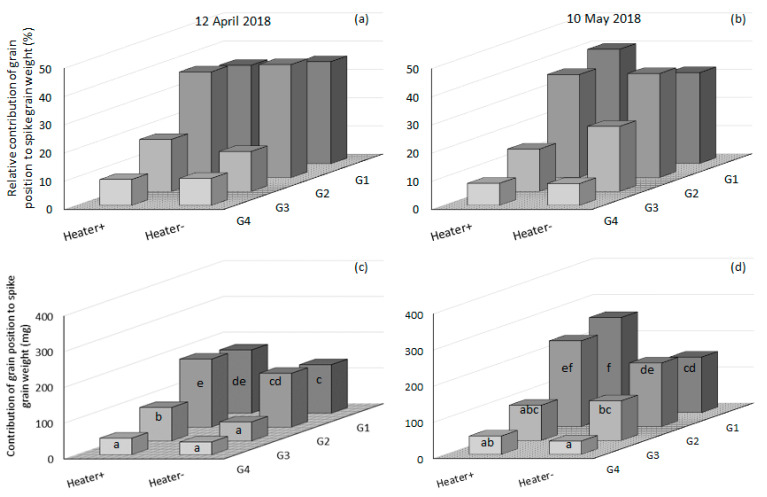
The relative contribution of grain at each position of the spikelet (grain position) to spike grain weight in Wyalkatchem sown in mid-April (12 April 2018 (**a**)) and mid-May (10 May 2018 (**b**)) sampled from heated and frosted non-heated plots. Absolute spike grain weights for the same sowing dates are shown on (**c**,**d**) with significance (*p* < 0.05) noted by letters with each sowing date compared separately. Relative contributions are calculated from the predicted means of absolute spike grain weights (n = 45 spikes per data column).
