# Supplementary material for: Estimating Effects of Radiation Frost on Wheat Using a Field-Based Frost Control Treatment to Stop Freezing Damage
Source: Genes (Basel). 2022 Mar 24;13(4):578. doi: 10.3390/genes13040578 (PMC9031182; doi:10.3390/genes13040578)
Supplement: Supplementary file 1 [file genes-13-00578-s001.zip › genes-1609339-supplementary.pdf]

Supplementary Material

Supplementary Figure S1.

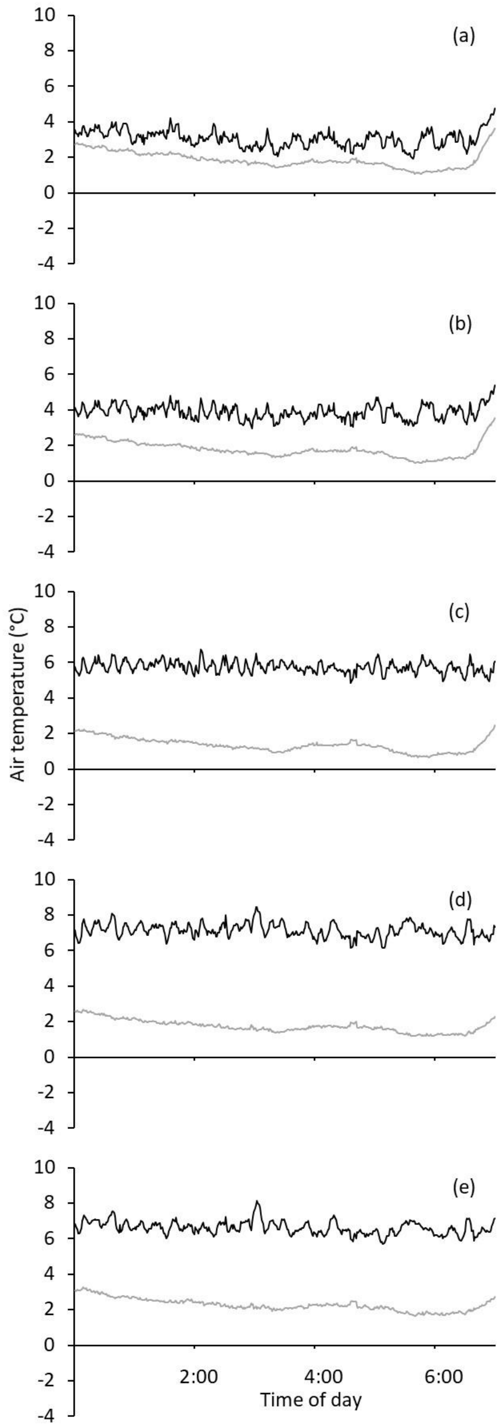

Figure S1. Average minute air temperature at 1000 (a), 800 (b), 600 (c), 400 (d) and 200 mm (e) above ground level (AGL) for the Heated plots (solid line) and adjacent non−heated plots (grey line) for frost events in July to October 2018.

Supplementary Figure S2.

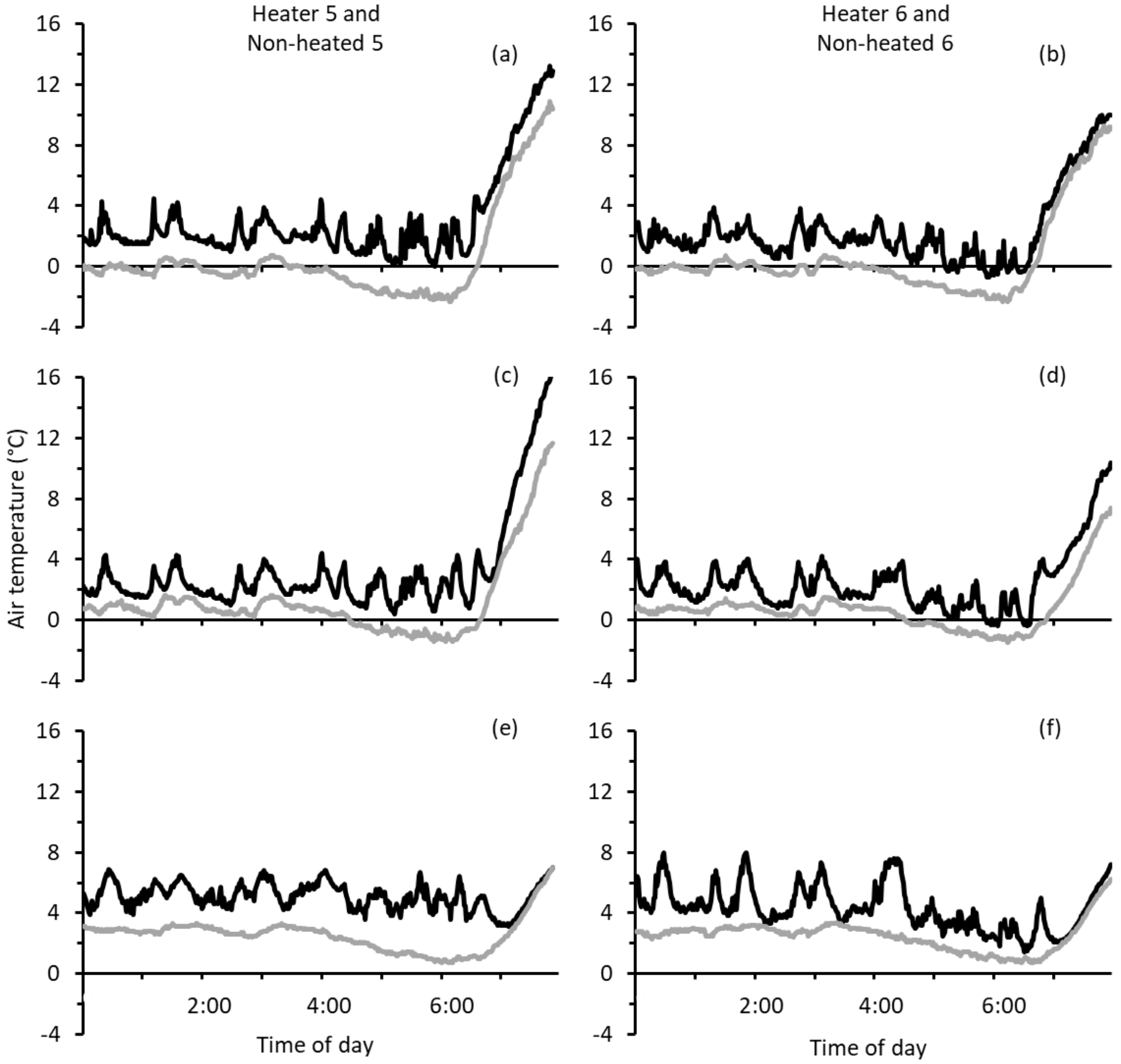

Figure S2. Air temperature at 800 (a and b), 400 (c and d) and 200 mm (e and f) AGL for the Heated plot (solid line) and adjacent non−heated plot (grey line) on 6 September 2019. Data is from Heater 5 and Non-heated plot 5 (a, c, and e) and Heater 6 and non−heated plot 6 (b, d, and f).

Supplementary Figure S3.

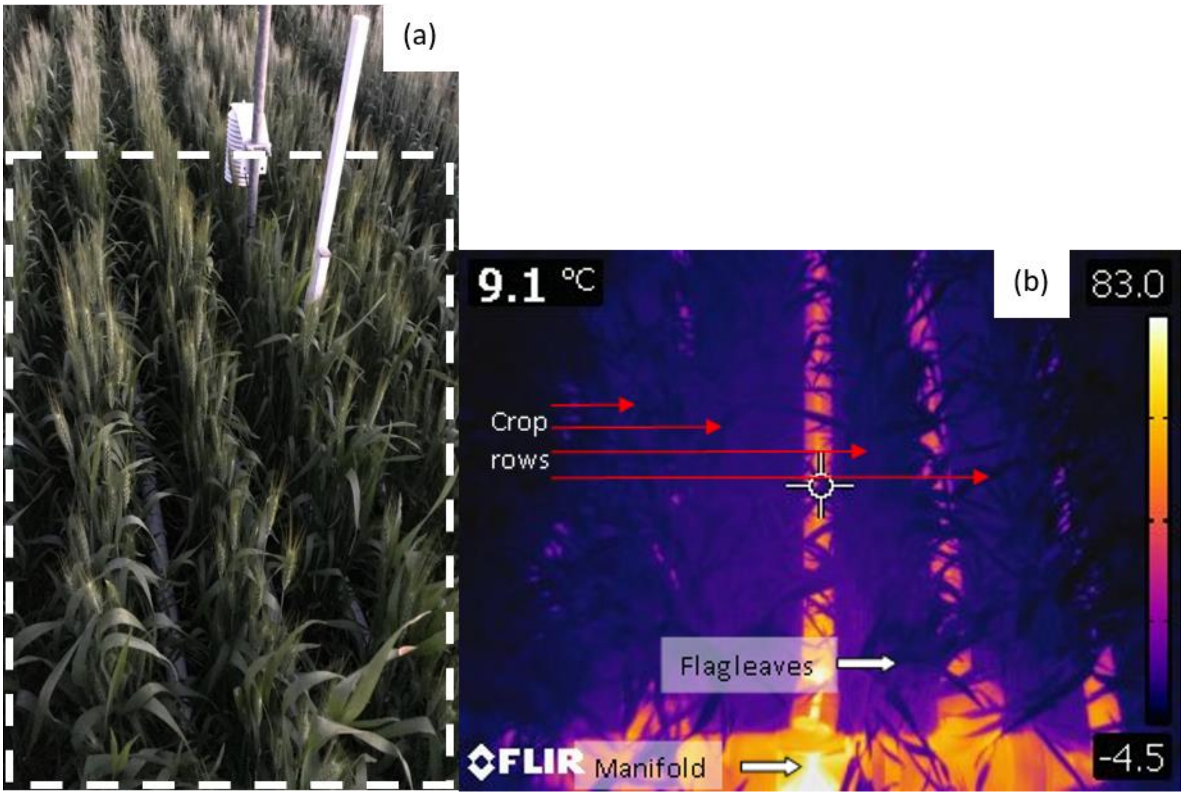

Figure S3. The diesel heater heating a field plot during a frost event at Dale August 18th, 2019 (a) digital photograph, (b) thermal image. In (a) the heated area can be seen by the darker canopy (inside dashed line box) which indicated that the heat kept most of the frost (white dew on the awns and spikes seen in the background) off the plants, i.e. prevented symptoms of plant frost exposure. In (b) the thermal image shows the warm PVC pipe and the colder canopy around the edge of the heated plot.

Supplementary Figure S4.

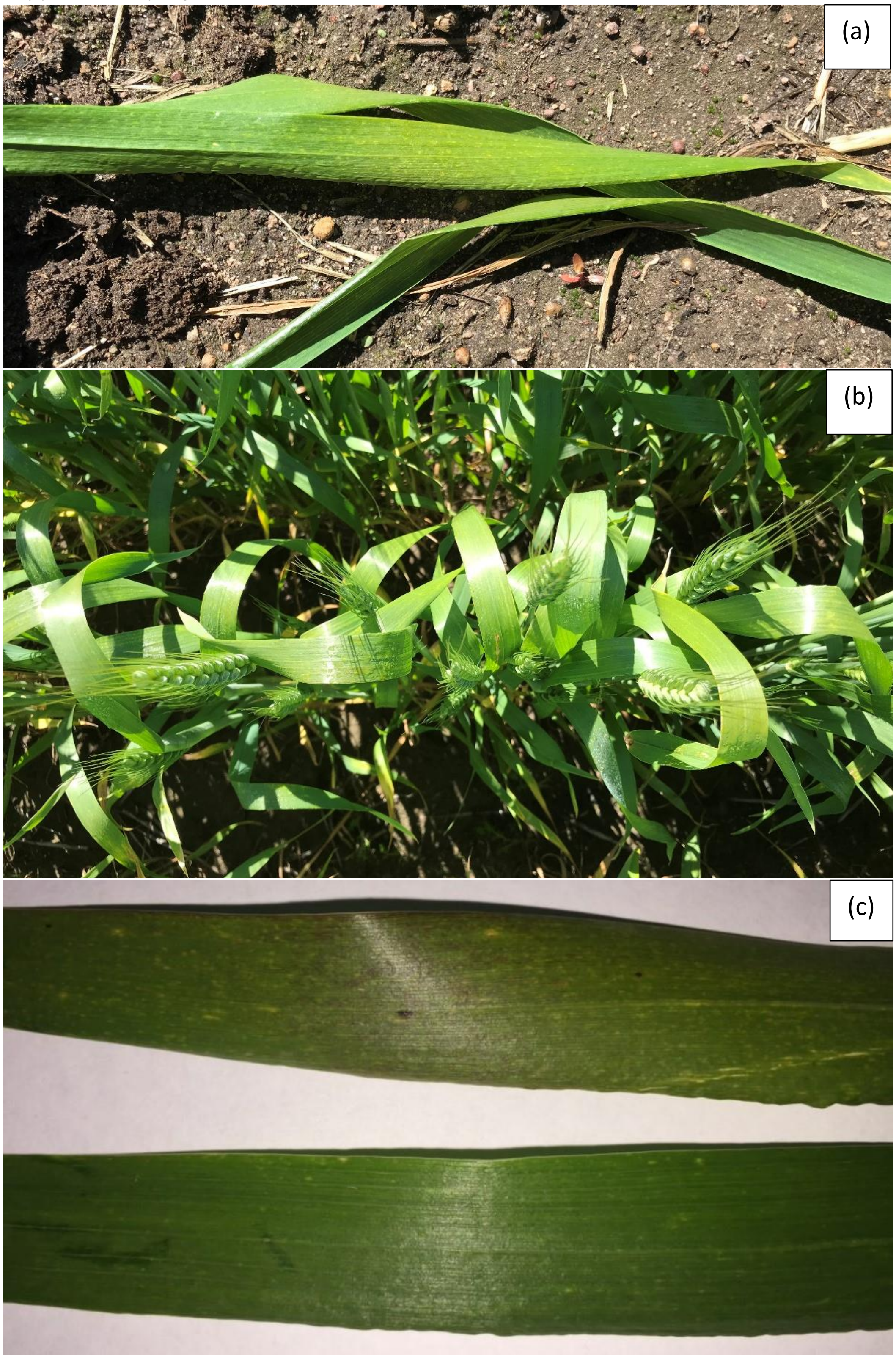

Figure S4. (a) Freezing damage to wheat flag leaves at Dale 6 September 2019. (b) The bleaching and plant tissue damage is isolated to sections of the flag leaves where they were horizontally orientated and cold dew pooled and froze there. Freezing damage presents itself bronze patches on leaves a few hours after the frost, this remains on the leaves for ~1−3 days.

Supplementary Figure S5.

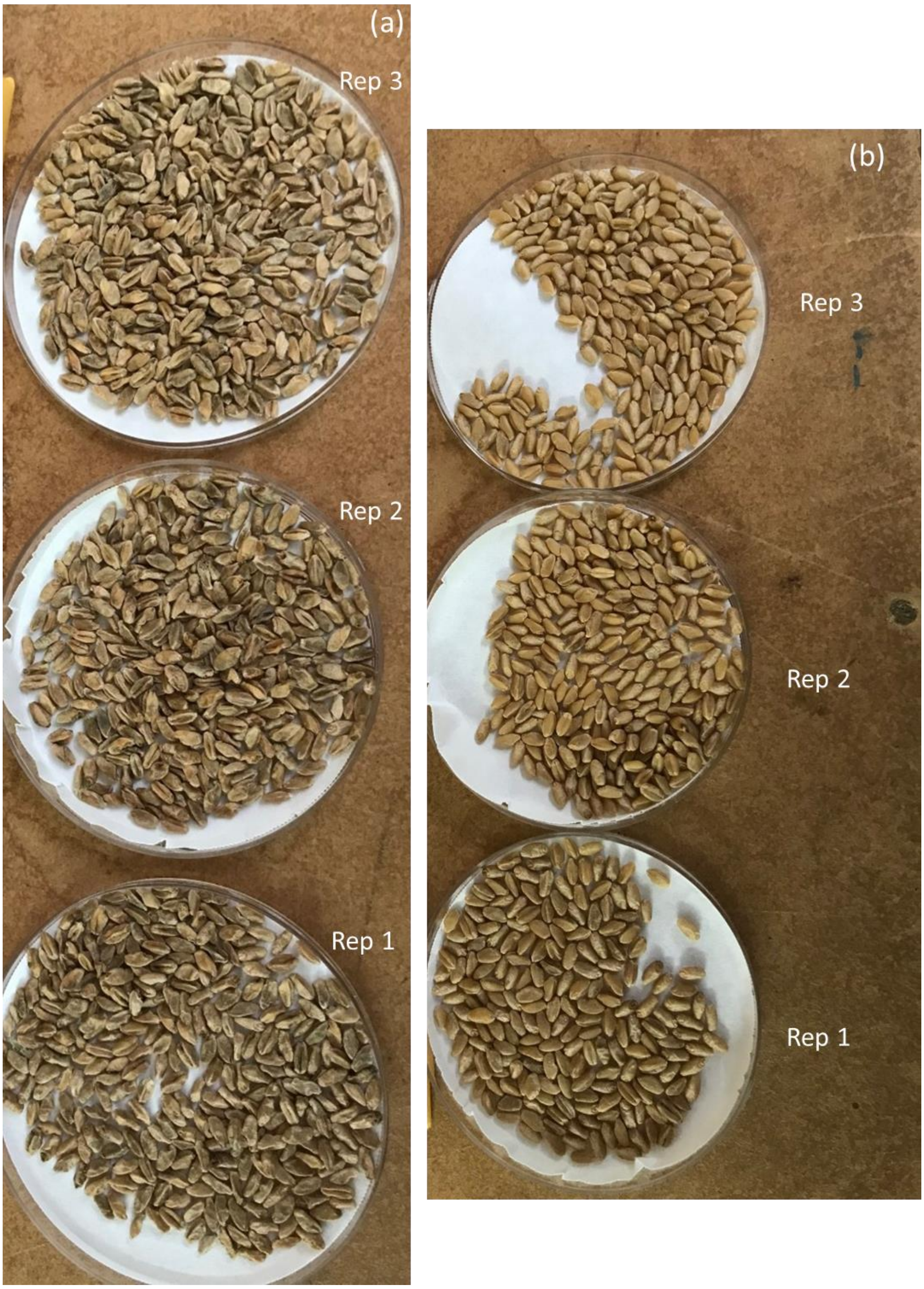

Figure S5. Images of grain samples from unheated plots harvest cuts ToS 1 (a) and ToS 5 (b) 2018. (a) shows clear evidence of grain damage due to frosts occurring at the grain filling stage (Zadoks growth stage Z70−77 from 7 to 14 August 2018) (Fig. 3a and Fig. 6a). This resulted in pinched and shrivelled grain like that seen in Cromey et al. 1998. (b) shows large grain size in unheated plots, despite moderate levels of flowering frost damage at (60% in heated) (Table 1). There were no frost events during grain filling stage (Figure 3a).
